# Dorzagliatin: A Breakthrough Glucokinase Activator Coming on Board to Treat Diabetes Mellitus

**DOI:** 10.7759/cureus.65708

**Published:** 2024-07-29

**Authors:** Ramya Raju, Indumathi Prabath, Indumathi Chandrasekaran, Sathyanarayanan Varadarajan

**Affiliations:** 1 Department of Pharmacology, Sri Ramaswamy Memorial (SRM) Medical College Hospital and Research Centre, SRM Institute of Science and Technology, Chengalpattu, IND; 2 Department of Pharmacology, Sri Venkateshwaraa Medical College Hospital and Research Centre, Puducherry, IND

**Keywords:** glucose sensitizers, glucose homeostasis, glucokinase, diabetes mellitus, dorzagliatin

## Abstract

Dorzagliatin, an innovative dual-acting allosteric oral glucokinase activator that targets glucose homeostasis and insulin resistance, has gained approval for treating type 1 diabetes mellitus (T1DM) and type 2 diabetes mellitus (T2DM). The effectiveness of existing antidiabetic treatments in enhancing beta cell (β-cell) activity is restricted. Currently, there are no satisfactory medications available to address the fundamental deficiency in glucose sensing for glucokinase-maturity-onset diabetes of the young (GCK-MODY), which is caused by mutations in the glucokinase gene; researchers have embarked on glucokinase activators. Dorzagliatin enhances the affinity of glucokinase for glucose and glucose-sensing capacity, improves β-cell function, and reduces insulin resistance. Two phase 3 studies, an adjunct trial of dorzagliatin with metformin for T2DM patients and a monotherapy trial for drug-naïve T2DM patients, are key clinical trials that have shown a favorable safety and tolerability profile. They also demonstrated a rapid, sustained reduction in glycated hemoglobin (HbA1c) and a significant decrease in postprandial blood glucose. This review will summarize the substantial clinical evidence supporting the safety and efficacy of dorzagliatin in treating diabetes mellitus (DM) and clarify the molecular mechanisms underlying its action.

## Introduction and background

Diabetes mellitus (DM) is a globally prevalent metabolic disorder characterized by high blood glucose levels and associated vascular complications. It develops due to the improper functioning of pancreatic beta cells (β-cells), resulting in insufficient insulin production or insulin resistance. India has often been referred to as the diabetes capital of the world, with the prevalence of diabetes being 9.3%, which accounts for almost 77 million people affected by it, next only to China. By the year 2045, it is expected that 134 million people will be affected by diabetes. Besides causing an increased risk of cardio- and cerebrovascular complications, diabetes also leads to renal failure and visual impairment. It has been reported that there have been 3 million deaths due to diabetic renal complications in 2019, despite the available medical treatment [1]. 

The quest for a revolutionary antidiabetic medication commenced with the discovery of insulin by Banting and Best in 1921. This breakthrough paved the way for many antidiabetic drugs targeting various insulin and glucose metabolic pathways, culminating in the approval of various distinct drugs. Despite the plethora of drugs available, there still exists a need for improvement of glycemic control and prevention of diabetic complications and drug-related adverse effects in the diabetic population. Additionally, it has been revealed that the global diabetes population has higher death rates with a twofold (95% confidence interval: 1.37-2.64) increase during coronavirus disease 2019 (COVID-19) infections, underscoring the need for more effective antidiabetic medications [2]. It might also be pertinent to understand that the presentation of diabetes and its complications are also different between Asians and Europeans, possibly due to genetic and epigenetic variations. 

Individuals with glucokinase-maturity-onset diabetes of the young (GCK-MODY) exhibit decreased β-cell glucose sensitivity and compromised alpha cell (α-cell) glucose sensing due to inactivating mutations in the glucokinase (GCK) gene [3,4]. There isn't yet a glucose-lowering drug that effectively addresses the fundamental problem of insufficient glucose sensing in GCK-MODY. Dorzagliatin is a groundbreaking, dual-acting allosteric activator of GCK, the first of its kind, which binds directly to a pocket distal to the active site of GCK [3]. This reduces the threshold for glucose-stimulated insulin secretion and enhances GCK's affinity for glucose, causing earlier and more robust insulin release in response to glucose aiding in overall glucose homeostasis [5,6]. This review focuses on the mechanism of action, trial evidence, and safety outcomes from the recently published literature on dorzagliatin.

## Review

Data collection and search strategy

Keywords such as dorzagliatin, glucokinase activators, glucose homeostasis, glucokinase physiology, and glucokinase modulators were used to gather articles from the Cochrane Clinical and PubMed/MEDLINE databases. For the review, articles explaining the dorzagliatin functions in type 2 DM (T2DM) and diabetic renal diseases were included. PubMed and American Diabetes Association webpages were accessed to retrieve the clinical trial information and data on the pharmacology of dorzagliatin.

Glucokinase activators (GCKAs) as antidiabetic medication

Kinases are ubiquitous enzymes involved in the phosphorylation of various biological substrates. They are classified according to the substrate they phosphorylate, namely, protein kinases, carbohydrate kinases (hexokinases), and lipid kinases (adipokines). GCK, a subset of hexokinases, have a major role in regulating insulin release triggered by glucose in pancreatic β-cells and managing the hepatic uptake of glucose and glycogen synthesis [4]. They are not only present in the pancreas and liver but also expressed in α-cell and delta cell of the pancreas, entero-endocrine cells, neurons, and cells in the anterior pituitary [7,8].

Mechanism of action of GCK

**Figure 1:**
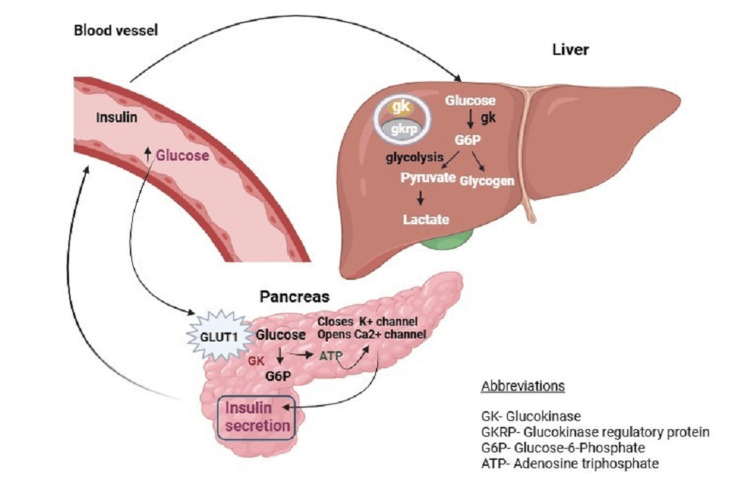
Physiological role of glucokinase in insulin secretion Developed by authors using the BioRender website Reference: [9]

GCK are present inside the liver complexing with the GCK regulatory protein (GCKRP). The GCKRP sequesters GCK when glucose levels are low and dissociates from glucose in the presence of raised glucose levels. It activates the GCK, inducing the phosphorylation of glucose into glucose-6-phosphate resulting in the release of insulin from the pancreas as well as glucose uptake and glycogen synthesis in hepatic cells as demonstrated in Fig. 1 [9].

Conformational changes modulate the functional properties of GCK, with the super-open state being inactive and the open and closed states being active. GCK can remain in the two high-affinity conformations, the closed and open forms, by combining GCKA and GCK to prevent GCK from changing from its conformation to the super-open forms [8,9]. This sustains the depolarization in the pancreatic β-cells, facilitating the opening of calcium channels and promoting insulin release [6].

**Figure 2:**
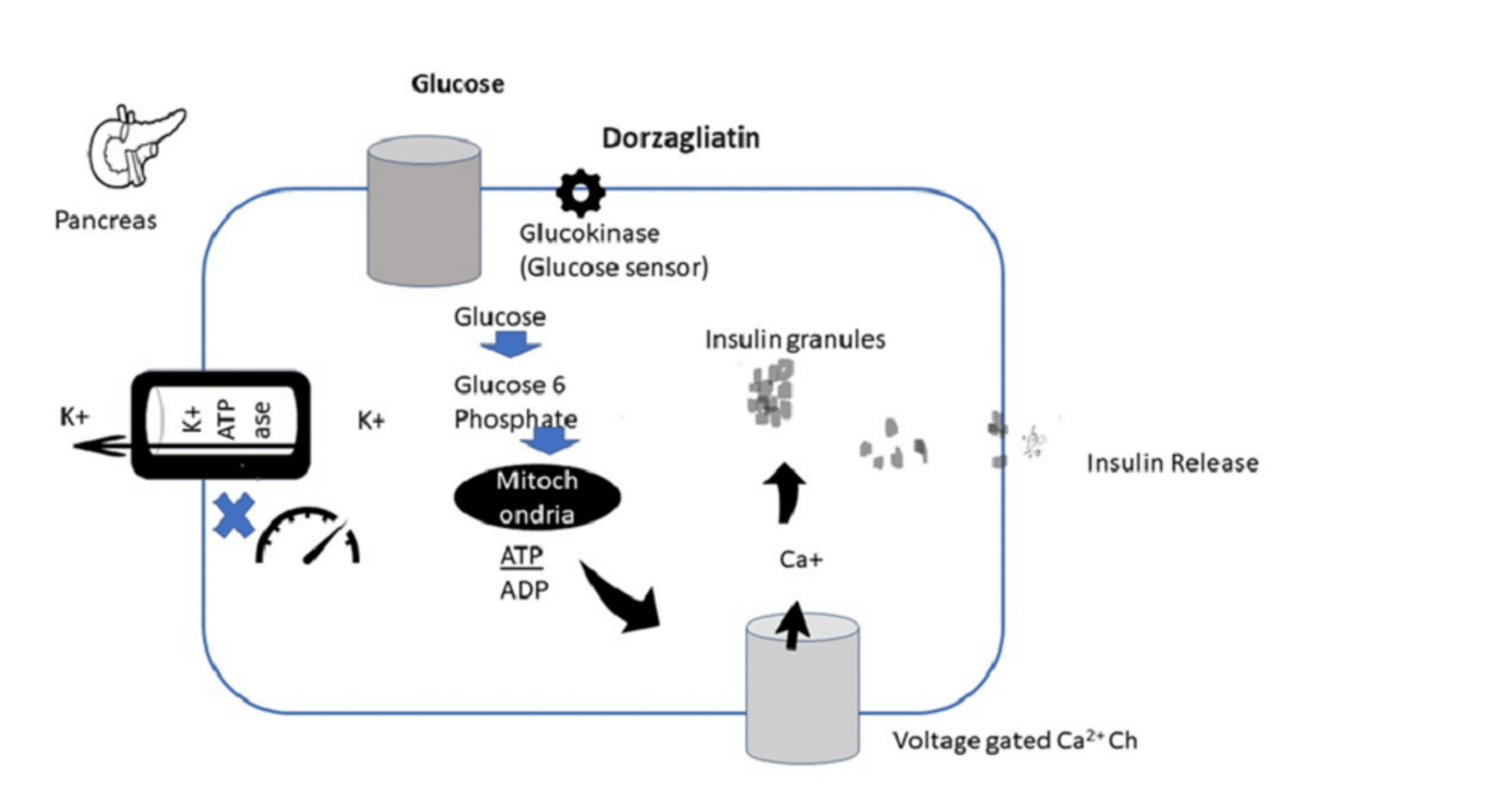
Intracellular mechanisms of glucokinase in pancreatic beta cells Developed by authors Reference: [6] ATP: adenosine triphosphate; ADP: adenosine diphosphate

GCK regulates insulin secretion and release by controlling glycolytic and oxidative adenosine triphosphate (ATP) production, thus closing the potassium (K+) channels and causing gradual depolarization of the pancreatic cell. Upon reaching the threshold membrane potential, the L-type calcium (Ca2+) channels open, leading to insulin release through the activation of various signaling pathways, including those involving Ca2+, cyclic adenosine monophosphate (cAMP), inositol-3-phosphate, and protein kinase C, as depicted in Fig. 2 [6].

It is believed that high glucose concentrations independently trigger GCK expression in β-cells, making them more sensitive to glucose-stimulated insulin release and biosynthesis [10]. Based on their mechanistic role, GCKAs have been tried as a potential antidiabetic medication since 2001. Over 20 GCKAs have been tried, and, among them, one has reached its culmination in 2022, namely, dorzagliatin [11].

Preclinical evaluation and genesis of the GCK glucose sensor

In the Pancreas

It was first discovered in 1927 that D-glucose plays a significant role in controlling blood sugar levels. It was shown that injecting minimal amounts of D-glucose into a dog's pancreaticoduodenal artery might lower its overall blood sugar levels [12,13]. Significant advancements in the research were made when it was discovered that D-glucose stimulates insulin secretion from the isolated perfused rat pancreas and insulin secretion in conjunction with glucose metabolism using portions of the rabbit pancreas [14,15]. GCK is a hexokinase 4 enzyme, identified by Matschinsky and Ellerman in 1968 [16]. The discoveries by Dean and Matthews in 1970 and Meissner and Schmelz in 1974 contributed to the understanding that GCK activity is linked to insulin release as glucose reduces the β-cell membrane potential [17,18]. Ashcroft and colleagues have shown that glucose triggers the closure of K+ ATP channels in pancreatic β-cells, indicating that the cellular energy state is crucial for linking glucose stimulation to insulin secretion [13,19].

In a study, transgenic mice exhibiting a twofold increase in hexokinase activity, specifically in pancreatic β-cells, were developed [4]. This enhancement causes isolated pancreatic islets to secrete insulin more quickly in response to glucose, increases serum insulin levels in vivo, and lowers the blood glucose levels of these transgenic animals by 20-50%, compared to the control [20]. Mice with one functioning GCK allele have a reduced β-cell response to glucose and become hyperglycemic when the mouse GCK gene is inactivated. On the other hand, mice that lack GCK entirely are born with severe diabetes and usually die. Mice with glucose levels ranging from normal to mildly diabetic that have limited expression of GCK in their β-cells survive [21-24]. GCKAs enhanced hepatic glucose intake, decreased blood glucose levels, and enhanced glucose tolerance test outcomes. GCKA drugs were found by Grimsby and colleagues [25]. The important roles of magnesium ATP and 5'-AMP as metabolic coupling variables in the glucose-induced stimulation of insulin secretion were highlighted in a simple computational model for GCK-based β-cell glucose sensing [13,26,27].

In the Liver

The standard Cre-Lox recombination (Cre-LoxP) gene is a tyrosine site-specific recombinase that recognizes and binds to specific DNA sequences known as loxP sites [28]. It deletes DNA segments between two sites of loxP. This is a commonly utilized technology for gene editing in mammals. A method for reducing GCK expression in mouse livers was examined. The mice appeared normal at birth but developed elevated fasting blood glucose levels as they aged. After six weeks, they exhibited impaired glucose tolerance and hyperglycemia. Higher levels of intracellular glucose 6-phosphate, glycogen, and L-pyruvate kinase activity were seen in a different group where there was an increase in hepatic GCK expression. These findings suggest that GCK overexpression may directly increase glycolysis and glycogen synthesis in vivo [28-31].

Evidence from clinical trials

Phase 1: Single Ascending Dose Study

The randomized phase 1 placebo-controlled clinical trial of HMS5552 (NCT01952535) was conducted at Zhongshan Hospital, China, to establish the safety profile, absorption, distribution, metabolism, and excretion (ADME) properties, and target effects in healthy volunteers (HV). Participants with a body mass index between 18 and 24 kg/m^2^, aged 18 and 45 years, with normal physical conditions and laboratory parameters including electrocardiogram, serology, and urinalysis, were recruited. Six doses of HMS5552, such as 5, 10, 15, 25, 35, and 50 mg, were tested among 60 participants, including 31 males and 29 females. Pharmacokinetic and pharmacodynamic (PD) assessments were performed in fasting and fed conditions. PD parameters were percentage change in glucose, average glucose area under the curve (AUC), and radio-immuno-based estimation of insulin. None of the participants encountered any serious adverse events except for six mild drug-related side effects such as dizziness, palpitation, sweating, and proteinuria. Fifty milligrams was determined to be the highest tolerated dose with the best safety profile and assessed and graded using the Common Terminology Criteria for Adverse Events version 4.02. This medication demonstrated a dose-dependent decrease in blood sugar with a 5-50 mg dosage range [32].

Phase 2: Clinical Trial

A multicentric phase 2 randomized clinical trial (NCT02561338) was conducted with dorzagliatin at four different regimens such as 75 mg once daily (OD), 100 mg twice daily (BD), 50 mg BD, and 75 mg BD for 12 weeks along with an oral placebo among T2DM patients. This study was conducted at 22 trial sites in China, recruiting both genders of patients. Study participants were either treatment naïve or on treatment with oral antidiabetic agents whose glycated hemoglobin (HbA1c) level should be within the parameters of 7.5-10.5%. Those patients with glomerular filtration rates less than 60 ml/min and elevated systolic and diastolic blood pressure of more than 160 and 100 mmHg, respectively, were excluded. Improvement in HbA1c was considered the primary endpoint. Patients were allocated into four groups in a 1:1:1:1 ratio through permuted block randomization. During the four-week run-in phase, other antidiabetic medications were withdrawn and replaced with a placebo. Testing for insulin, fasting plasma glucose (FPG), and HbA1c was performed at baseline, every two weeks, and at the end of the therapy period. β-Cell function and postprandial plasma glucose were assessed at the beginning and conclusion of the treatment phase. Out of 619 screened patients, 258 were randomized into five groups: 53 in the placebo group, 53 in the 75 mg OD group, 50 in the 100 mg OD group, 51 in the 50 mg BD group, and 51 in the 75 mg BD group. The study found that after 12 weeks, dorzagliatin treatment led to a significant reduction in HbA1c levels by 0.44% (95% CI: -0.78 to -0.10) in the 50 mg BD group and 0.77% (-1.11 to -0.43) in the 75 mg BD group. Additionally, 22 participants (44%) in the 50 mg BD group (odds ratio: 3.70 (95% CI: 1.46-9.38)) and 22 (45%) in the 75 mg BD group (odds ratio: 4.33 (95% CI: 1.54-12.19)) achieved an optimal response with HbA1c levels below 7%. Those on dorzagliatin 50 mg BD and 75 mg BD had 2.99 (95% CI: 0.97-9.25) and 4.33 (95% CI: 1.43-13.13) times greater likelihood of reaching optimal HbA1c levels without experiencing hypoglycemia or weight gain. In homeostatic model assessment for insulin resistance (HOMA-IR), a significant improvement over placebo was documented only in the 75 mg BD group. It was evident that drug-naïve patients showed considerable change in HbA1c with all four regimens of dorzagliatin compared to patients on standard antidiabetic medications. Treatment-related mild side effects were noted. Around 6%, 4%, 6%, and 6% of patients in 75 mg OD, 100 mg OD, 50 mg BD, and 100 mg BD, respectively, reported having hypoglycemia, and all of these episodes were transient. It was concluded from the study that dorzagliatin exhibited dose-dependent efficacy and limited side effects during 12 weeks of therapy, and it was also declared that 75 mg BD would be the least effective dose for further clinical evaluation. Extrapolation of study results to different ethnic groups might be restricted as the study included only the Chinese population [33].

Phase 3: Clinical Trial Study of Early and Exploratory Development (SEED Study)

Drug-naïve Chinese patients with T2DM were assessed for the safety and effectiveness of dorzagliatin in a phase 3 SEED trial involving 40 sites. Within the trial, there was a 24-week double-blind, placebo-controlled phase; participants received 75 mg dorzagliatin BD during the 28-week open-label phase; after that, there was a one-week treatment-free follow-up. Participants were randomly assigned 2:1 to receive dorzagliatin or a placebo after a two-week screening period. At week 24, the dorzagliatin group showed an HbA1c decrease of 1.07% compared to the placebo group (estimated treatment difference: -0.57%; 95% CI: -0.79 to -0.36; P<0.001). An estimated treatment difference of 3.28 (95% CI: 0.44-6.11; P<0.05) was observed in β-cell function when dorzagliatin was compared to placebo homeostatic model assessment of β-cell function (HOMA2-β change: -0.72). From week 8 to week 24, subgroup analysis showed higher dorzagliatin homeostatic control rates (odds ratio: 3.60; 95% CI: 1.81-7.14; P<0.001), particularly in patients with baseline HbA1c ≤8.0%. Dorzagliatin significantly reduced postprandial and fasting glucose levels throughout a 52-week period. In addition to acting quickly and not causing weight gain, the medication was well-tolerated. During the double-blind phase, there was only one case of mild hypoglycemia in each group, and a minimal range of adverse events was seen. The results showed that dorzagliatin improves early-phase insulin release and β-cell function, resulting in quick and stable glycemic control without raising the risk of dyslipidemia or liver damage [34].

Diabetes Remission Clinical Trial (DREAM) Extension Study

A nonpharmacologic observational clinical trial, extension of the SEED project, the DREAM study, included 69 patients in 2023 who met investigator-assessed glycemia objectives after 52 weeks of dorzagliatin medication. The study's primary goal was to determine the probability of diabetes remission following the cessation of dorzagliatin and without the need for hypoglycemic medications. The Kaplan-Meier approach was employed to determine this outcome. Its results demonstrated that after 52 weeks, the chance of diabetes remission had reached 65.2%. The primary endpoint showed that patients with a baseline HbA1c of less than 6.5% had a remission probability of 80.1% at week 52, which was greater than that for patients with an HbA1c of >6.5%. After 46 weeks of dorzagliatin therapy, individuals with a time in range (TIR) of 80% or above had a greater probability of remission than those with a TIR of less than 80% [35].

Phase 3: Clinical Trial Dorzagliatin Assessment for Weekly Normoglycemia (DAWN) Study

Patients with T2DM who were not adequately controlled with metformin alone (HbA1c levels between 7.5% and 10%) were recruited for a phase 3 randomized, double-blind, placebo-controlled study to assess the safety and effectiveness of dorzagliatin as an adjuvant medication. Patients were randomized in a 1:1 ratio to receive metformin (1500 mg/day) plus dorzagliatin (75 mg BD) or a placebo for 24 weeks, with a 28-week open-label phase in between. The study was carried out at 73 sites in China. HbA1c, FPG, and two-hour postprandial glucose (2h-PPG) were the primary endpoints. HOMA2-β and HOMA2-IR indices were utilized to evaluate β-cell function and insulin resistance. The group that received dorzagliatin plus metformin after 24 weeks demonstrated a drastic decrease in HbA1c of 1.02 percentage points, while the placebo group experienced a reduction of 0.36 percentage points. With an estimated treatment difference of -0.66 percentage points (95% CI: -0.79 to -0.53; P<0.001), the dorzagliatin group had a significantly greater HbA1c response rate of 44.4%. Furthermore, higher reductions in FPG and 2h-PPG levels were observed with dorzagliatin. There were also improvements in β-cell function and insulin resistance; the dorzagliatin group had a HOMA2-β change of 3.82 compared to 1.40 in the placebo group (estimated treatment difference: 2.43; 95% CI: 0.59-4.26; P<0.01). In the dorzagliatin group, the HOMA2-IR change was -0.17, while in the placebo group, it was -0.09 (estimated treatment difference: -0.08; 95% CI: -0.15 to -0.01; P<0.05). Through week 52, there was no increase in HbA1c. The coadministration of metformin and dorzagliatin resulted in mild hypoglycemia, which was well-tolerated. Further studies with larger sample sizes and longer-term safety would be needed to conclude the possibility of using dorzagliatin as an add-on therapy to metformin in improving glycemic control in T2DM [36].

Dorzagliatin in Kidney Disease

In a study with 17 Chinese participants, eight of whom had end-stage renal disease (ESRD) and nine were HVs, the pharmacokinetics of a single oral dose of dorzagliatin, 25 mg, were investigated. Part 1 results showing the AUC geometric mean ratio that is AUC from the administration time to the last measurable concentration (AUClast) or AUC to infinity (AUCinf) between ESRD patients and HVs surpassing 100% were required for part 2. Dialysis-free ESRD patients and healthy controls participated in part 1, while part 2 involved patients with varying renal impairment (RI) from mild to severe. Eligibility was assessed using the Modification of Diet in Renal Disease formula, unique to China by which estimated glomerular filtration rate (eGFR) categorizes the severity of RI. Normal renal function (eGFR ≥90 ml/min/1.73 m^2^), ESRD not yet on dialysis (eGFR <15 ml/min/1.73 m^2^), severe renal function (eGFR 15-29 ml/min/1.73 m^2^), moderate renal function (eGFR 30-59 ml/min/1.73 m^2^), and mild renal function (eGFR 60-89 ml/min/1.73 m^2^) were the eGFR categories. The participants were given dorzagliatin following an overnight fast and a predetermined meal. High-performance tandem mass spectrometry and liquid chromatography were employed. The side effects were relatively low. The pharmacokinetics of dorzagliatin were expected to be largely unaffected by RI, according to physiologically based pharmacokinetic (PBPK) modeling. As the renal clearance (CLr) predicted by the PBPK and observed clinical pharmacokinetic values of HV and ESRD ratios are 1.13 and 0.74, respectively, it shows that PBPK modeling could predict even a tiny proportion of renal excretion of dorzagliatin with accuracy. This prediction was confirmed by part 1 results, which showed that patients with diabetic kidney disease (DKD) did not require dose adjustments. Over a dosage range of 5-150 mg BD, dorzagliatin demonstrated a sizable safe safety margin, linear pharmacokinetics, and a predictable dose-response relationship. Consequently, at a dosage of 75 mg BD, a 30% increase in exposure AUC is not anticipated to have an impact on effectiveness or safety [37]. The major clinical aspects of significant dorzagliatin trials are presented in Table 1.

**Table 1:** Synopsis of clinical trials involving dorzagliatin RI: renal impairment; DKD: diabetic kidney disease; OD: once daily; BD: twice daily; HbA1c: glycated hemoglobin; 2h-PPG: two-hour postprandial glucose; FPG: fasting plasma glucose; HOMA2-β: homeostatic model assessment of β-cell function; HOMA2-IR: homeostatic model assessment for insulin resistance

S. no.	Clinical trial	The phase of the study	Sample size	Dose (mg)	Comparator	Primary effect	Adverse effects
1	Xu et al. [32]	Phase 1a	60	5, 10, 15, 25, 35, and 50 mg	Placebo	Tolerated dose was declared as 50 mg	No serious side effects
2	Zhu et al. [33]	Phase 2	258	Oral dorzagliatin at one of four doses (75 mg OD, 100 mg OD, 50 mg BD, or 75 mg BD)	Placebo	Dose-dependent reduction in HbA1c and 75 mg was found to be the least effective dose for further study	Minimal side effects
3	Chen et al. [34]	Phase 3	463	75 mg BD	Placebo	Reduction in HbA1c, 2h-PPG, and FPG levels and increase in HOMA2-β	No severe hypoglycemia and no drug-specific aberrant liver function
4	Chen et al. [36]	Phase 3	767	75 mg BD as an add-on to metformin 1500 mg	Placebo	Improvement in HOMA2-β and HOMA2-IR and reduction in FPG, 2h-PPG, and HbA1c levels	Minimal side effects
5	Miao et al. [37]	RI study, DKD vs healthy volunteers	17	25 mg OD	None	No dose adjustment is needed in DKD	Minimal side effects

Dorzagliatin Approval

While most GCKAs faced challenges in phase 2 clinical trials due to their antidiabetic potential, dorzagliatin stands out as an exception. MK-0941 was unsuccessful because of high hypoglycemia rates and limited efficacy. The hepatic GCK stimulator PF-04991532 showed a 0.7% reduction in HbA1c over 12 weeks but was halted due to toxic metabolites, a problem that also affected the dual activator piragliatin. In contrast, Hua Medicine reported in December 2020 that two phase 3 trials of dorzagliatin, SEED (HMM0301) for drug-naïve T2DM patients and DAWN (HMM0302) for those tolerant to metformin, resulted in significant decreases in 2h-PPG and HbA1c levels. Additionally, dorzagliatin enhanced β-cell function, decreased insulin resistance, and was well-tolerated with a good safety profile. It also exhibited synergistic effects when used with sitagliptin and empagliflozin in phase 1 studies. With its approval in China in September 2022, dorzagliatin is now prescribed both as a monotherapy and in combination with metformin for adults with T2DM. Considering the genetic and dietary parallels, dorzagliatin is viewed as a hopeful option for glycemic management in the Indian diabetic population.

Advantages and disadvantages of dorzagliatin

Merits of Dorzagliatin

Dorzagliatin reduces the risk of hypoglycemia, liver damage, and weight gain while improving glycemic control by using the glucose-sensing capabilities of GCK. Dorzagliatin has successfully and safely decreased HbA1c levels in patients who were previously unresponsive, in contrast to other oral antidiabetic medications like thiazolidinediones, dipeptidyl peptidase-4 (DPP-4) inhibitors, α-glucosidase inhibitors, and sodium-glucose cotransporter-2 (SGLT2) inhibitors. It can be used safely in DKD, including ESRD, without changing the dose. Dorzagliatin can be used in conjunction with other diabetes drugs to offer prolonged glycemic control, which is essential for controlling chronic diabetes. Its specific mechanism targets GCK, lowering the risk of both hypoglycemia and hyperglycemia. And also, by maintaining the function of pancreatic β-cells, it may lessen the long-term effects of T2DM and slow its progression.

Disadvantages With GCKAs

Excess fat deposition in the liver and elevated triglycerides and blood pressure thrust more burden on T2DM patients who were already prone to metabolic complications. Fading of GCK activity over the years decreases the antidiabetic efficacy of GCKAs [38]. Though dorzagliatin exhibited a similar percentage with the control group and is relatively safe, it still needs extensive studies and real-world population treatment to conclude the adverse reactions of the drug.

Future scope of research

Further research is needed to support the theory that glucose homeostasis and hormone release may also be influenced by the ability of GCK to sense glucose at the level of neurons, adrenal glands, the gut, and the anterior pituitary [13].

Neurons

According to study reports, pro-opiomelanocortin, neuropeptide Y, and γ-aminobutyric acid-containing hypothalamic neurons, along with the norepinephrine neurons of the locus ceruleus, express GCK mRNA. Four selective GCK inhibitors were found to inhibit intracellular Ca2+ oscillations in glucose-excited neurons and to stimulate them in glucose-inhibited neurons. These findings led researchers to explore the mechanism of GCK action further [13,39].

Hypoglycemia in the nucleus of the vagus nerve's tractus solitarius activates excitatory neurons that express glucose transporters (GLUT2), and GCK mediates this process. An increase in AMP and a decrease in blood glucose levels promote the inhibition of K+ leak current and the activation of AMP-dependent protein kinase. These processes result in cellular hyperpolarization and enhanced afferent electrical activity. Consequently, the importance of GCK as a glucose sensor expands the scope of this fascinating field of study [13,40].

Hypothalamus

Energy homeostasis mediated by GCK is located at the hypothalamus arcuate nucleus. A study in male Wistar rats with elevated GCK activity in the arcuate nucleus demonstrated an increase in food intake and excess weight gain. Similar effects were replicated when the K+ ATP channel was blocked by glibenclamide, which was administered intra-arcuately. Additionally, intra-arcuate injection of diazoxide, a K+ ATP channel activator, decreased GCK activity. Also demonstrated is the binding of GCK to GCK inhibited during fasting, as the GCK activity gets accelerated during fasting and the binding increased by hyperglycemia has expressed an option to evaluate the role of GCK in healthy low body mass index patients and low weight patients [41].

According to the hypothesis, GCK is important for glucose signaling in the neurons of the ventromedial hypothalamic nucleus (VMN), which is the center for feeding and glucose homeostasis regulation. Researchers looked at mice whose GCK gene in the VMN's Sf1 neurons was genetically inactivated to learn more about the function of GCK in VMN glucose sensing and physiological regulation. This study used whole-cell patch clamp studies of brain slices to study the function of GCK in glucose sensing. The results demonstrated that GCK expression prevented both gender-specific glucose-excited and glucose-inhibited Sf1 neurons from sensing glucose [42]. Further studies would be required to confirm the GCK expression of VMN neurons in glucose-sensing ability.

Confirmatory studies regarding the safety and efficacy of dorzagliatin are yet to be carried out in patients with cardiovascular disease, in obese patients, and in combination with other standard treatment regimens. Dorzagliatin can also be tested as an early intervention, for the effects on liver enzymes, drug-drug interactions, and real-world effectiveness.

Adherence and pharmacoeconomic evaluation

A prospective follow-up study on patients who achieved stable glucose levels and HbA1c after long-term treatment with dorzagliatin was carried out, and interestingly, there seems to be a 52% (American Diabetes Association guidelines 2021) to 65% (Kaplan-Meier remission probability) of diabetes remission during 52 weeks among Chinese T2DM patients [35]. If these results are replicated in large-scale studies of five years or longer duration, it could tremendously benefit the diabetic population. The annual expenditure for antidiabetic treatment for an adult is USD 35,219 in 2022 for the most cost-effective regimen [43]. A systematic review found that medication adherence varied between 38.5% and 93.1%. Non-adherence to antidiabetic medication in this study was attributed to depression and medication costs; the two factors were consistently associated predictors of non-adherence [44]. Dorzagliatin by way of significant time-in range and disease remission can have a huge pharmacoeconomic implication for 537 million adults worldwide provided its safety is established [1].

## Conclusions

Dorzagliatin is a novel and potent drug that can be administered to treat T2DM. In hepatocytes and pancreatic β-cells, GCK is the primary target as it is necessary for glucose sensing and homeostasis. By modifying GCK activity, it improves insulin sensitivity. Although it is generally well-tolerated, according to early studies, there are certain research gaps and challenges, such as insufficiency of long-term safety data. Moreover, in subsequent clinical evaluations, the efficacy of combination therapy and its cost-effectiveness need to be addressed in various global populations.
